# Comparative investigation on heterotrophic denitrification driven by different biodegradable polymers for nitrate removal in mariculture wastewater: Organic carbon release, denitrification performance, and microbial community

**DOI:** 10.3389/fmicb.2023.1141362

**Published:** 2023-02-20

**Authors:** Yuna Feng, Lu Wang, Zhendong Yin, Zhengguo Cui, Keming Qu, Dawei Wang, Zhanying Wang, Shengmin Zhu, Hongwu Cui

**Affiliations:** ^1^National Experimental Teaching Demonstration Center for Aquatic Science, National Demonstration Center for Experimental Fisheries Science Education, Shanghai Ocean University, Shanghai, China; ^2^Key Laboratory of Sustainable Development of Marine Fisheries, Ministry of Agriculture and Rural Affairs, Yellow Sea Fisheries Research Institute, Chinese Academy of Fishery Sciences, Qingdao, China; ^3^Marine Life Research Center, Laoshan Laboratory, Qingdao, China; ^4^College of Chemistry and Chemical Engineering, Ocean University of China, Qingdao, China

**Keywords:** solid carbon source, slow-release, nitrate removal, mariculture wastewater, low C/N

## Abstract

Heterotrophic denitrification is widely studied to purify freshwater wastewater, but its application to seawater wastewater is rarely reported. In this study, two types of agricultural wastes and two types of synthetic polymers were selected as solid carbon sources in denitrification process to explore their effects on the purification capacity of low-C/N marine recirculating aquaculture wastewater (NO_3_^−^-N 30 mg/L, salinity 32‰). The surface properties of reed straw (RS), corn cob (CC), polycaprolactone (PCL) and poly3-hydroxybutyrate-hydroxypropionate (PHBV) were evaluated by Brunauer–Emmett–Teller, Scanning electron microscope and Fourier-transform infrared spectroscopy. Short-chain fatty acids, dissolved organic carbon (DOC), and chemical oxygen demand (COD) equivalents were used to analyze the carbon release capacity. Results showed that agricultural waste had higher carbon release capacity than PCL and PHBV. The cumulative DOC and COD of agricultural waste were 0.56–12.65 and 1.15–18.75 mg/g, respectively, while those for synthetic polymers were 0.07–1.473 and 0.045–1.425 mg/g, respectively. The removal efficiency of nitrate nitrogen (NO_3_^−^-N) was CC 70.80%, PCL 53.64%, RS 42.51%, and PHBV 41.35%. Microbial community analysis showed that Proteobacteria and Firmicutes were the most abundant phyla in agricultural wastes and biodegradable natural or synthetic polymers. Quantitative real-time PCR indicated the conversion from nitrate to nitrogen was achieved in all four carbon source systems, and all six genes had the highest copy number in CC. The contents of medium nitrate reductase, nitrite reductase and nitrous oxide reductase genes in agricultural wastes were higher than those in synthetic polymers. In summary, CC is an ideal carbon source for denitrification technology to purify low C/N recirculating mariculture wastewater.

## Introduction

Compared to the traditional aquaculture system, the recirculating aquaculture system (RAS) plays an increasingly important role in the production model of aquaculture because of its small floor area, controllable breeding conditions, potential to effectively reduce the waste of water resources and ability to realize the advantages of high-density cultivation (Zou et al., 2018). Wastewater treatment is a key issue for RAS, and ammonia nitrogen (NH_4_^+^-N) and nitrite nitrogen (NO_2_^−^-N) produced by residual bait and feces were toxic to aquaculture organisms. Therefore, NH_4_^+^-N and NO_2_^−^-N must be transformed into nitrate nitrogen (NO_3_^−^-N) having less toxicity through biological filters. With NO_3_^−^-N pollution in water getting increased attention, numerous studies have shown that NO_3_^−^-N in marine RAS has become one of the major sources of water environment pollution, and the accumulation of NO_3_^−^-N will have long-term negative effects on aquaculture organisms (Davidson et al., 2017). The concentration of NO_3_^−^-N is usually controlled by water exchange, which not only increases the wastewater generation but also threatens the ecological environment (Martins et al., 2010). Denitrification technology is an effective way to reduce NO_3_^−^-N pollution in RAS wastewater (Johann et al., 2018). As with most mariculture wastewater, a low carbon-to-nitrogen ratio (C/N) is the main factor limiting the denitrification process because of its very low concentration of organic pollutants. Therefore, RAS wastewater treatment usually relies on the supplementation of additional carbon sources to ensure high efficiency and adequacy of denitrification (Zhao et al., 2012).

Common types of carbon sources in denitrification can be divided into water-soluble organic carbon and solid carbon. In practical applications, the addition of water-soluble organic carbon sources to the influent in the denitrification unit was studied earlier, mainly including alcohols such as methanol and ethanol (Fajardo et al., 2012), fatty acids such as acetate and propionic acid, and sugars such as glucose (Nyberg et al., 1996). However, relevant studies have found that water-soluble carbon source dosage is difficult to control and is prone to NO_2_^−^-N accumulation and other secondary pollution problems (De Schryver et al., 2008). Therefore, some biodegradable polymers have been studied extensively because of their good long-term denitrification effect and controllable effluent chemical oxygen demand (COD). Many water-insoluble but biodegradable natural material and synthetic biodegradable polymers (BDPs) can be used as carriers for microorganisms in wastewater (Epsztein et al., 2016). Agricultural wastes, such as reed straw (RS), straw (SA), and corn cob (CC) are reported to have a good nitrogen removal effect and low price compared to other carbon sources (Rosales et al., 2017). BDPs (such as polyhydroxy chain alkylate (PHA), poly3-hydroxybutyrate-hydroxypropionate (PHBV), polycaprolactone (PCL), polybutanediol succinate (PBS), etc.) have been extensively studied for their good resistance to impact loading, while they are not economical compared to natural plants (Zhu et al., 2015). Therefore, the selection of additional carbon source is critical for the complete denitrification, so as not to reduce the nitrogen removal efficiency of denitrification or bring secondary pollution.

At present, the relevant research with solid carbon resource addition mainly focuses on the treatment of urban groundwater and industrial wastewater, and found that the denitrification efficiency can be effectively improved. Jia et al. (2019) explored the feasibility of using wheat straw, apricot pits, and walnut shells for nitrogen removal from low C/N synthetic wastewater in constructed wetland, and found that the total nitrogen removal efficiency was as high as 66.75%–93.67%. Cui et al. (2022) used wastepaper to purify wastewater from sewage treatment plants, it was found that the denitrification efficiency could reach 94.17%. However, the solid carbon source-driven denitrification technology to purify marine recirculating aquaculture wastewater is rarely studied. The main pollutant in marine recirculating aquaculture wastewater is nitrate nitrogen. Different from other wastewater, it has the characteristics of low C/N and high salinity. These characteristics may affect the growth of denitrifying bacteria, which is not conducive to denitrification. It is not clear whether solid carbon sources used for freshwater denitrification can maintain their carbon source release capacity in mariculture wastewater. And whether the high salinity and low-C/N characteristics of mariculture wastewater will reduce denitrification efficiency or produce other concerns. In this study, two agricultural wastes (RS and CC) and two BDPs (PCL and PHBV) were selected as the additional carbon sources. The surface characteristics, carbon release performance, and denitrification capacity of each solid carbon source were comprehensively investigated by conducting carbon source release and nitrate removal experiments. Based on the research results, the best solid carbon source was selected, and the feasibility of the four selected carbon sources as additional carbon sources for denitrification was evaluated, providing a reference for the selection of carbon sources for denitrification treatment of marine recirculating aquaculture wastewater.

## Materials and methods

### Materials

Four external carbon sources were used in this experiment. Agricultural waste RS and CC were purchased from Henan Gongyi Hengrun Water Treatment Materials Co., Ltd. (China). Before the experiment, the agricultural waste was washed with deionized water and dried in a drying oven at 60°C until constant weight. PCL and PHBV were purchased from the Dongguan Camphor Wood Suyuan Plastic Raw Materials Business Department (China). The physical properties of the selected materials are presented in Table 1. The Brunauer–Emmett–Teller surface area, pore-volume, and pore size were obtained by using fresh carbon source materials placed in static tubes, pumped to vacuum and degassed with Micromeritics Tristar II 3020 (USA), and measured in liquid nitrogen after depressurization and cooling.

**Table 1:** The physical properties of the four types of carbon sources.

Carbon source	Appearance shape	Particle size (mm)	Brunauer–Emmett–Teller surface area (m^2^/g)	Pore volume (cm^3^/g)	Pore size (nm)
RS	Schistose	4–5	0.6388	0.001737	26.3734
CC	Schistose	4–5	0.2043	0.000479	10.9531
PCL	Cylinder	4–5	0.0487	0.000029	45.2805
PHBV	Pellet	4–5	0.0206	0.000028	3.2825

### Carbon source screening experiment

#### Carbon source release experiment

Add 40 grams of each carbon source into a 1,000 mL wide mouth bottle, and mixed with 800 ml natural seawater (salinity 31 ± 1 ‰, pH 7.72 ± 0.15, DO 5.56 ± 0.41, COD 2.32 ± 1.17). The bottle was then sealed with a plastic cap to prevent evaporation and contamination. The experimental temperature was 25°C and the experimental period was 30 days. The water in the bottle was collected every 2 days for determination of COD, TOC, and, DOC. Analysis of CODs was done according to the method described in the Standard Method for Examination of Water and Wastewater GB 17378.7-1998 (1999). TOC and DOC were measured using a TOC analyzer (Shimadzu, TOC-L) with an automatic sampler (Shimadzu, ASI-V). At the end of this experiment, the contents of short-chain fatty acids (SCFAs) in water were measured. SCFAs, including acetic acid (AC), propionic acid (PA), butyric acid (BA), and other types (isobutyric acid, valeric acid, isovaleric acid, and hexanoic acid) were measured by ion chromatography-mass spectrometry (Shimadzu GCMS QP2010-ULTRA, Japan). After each sampling, an equal amount of sterilized seawater was added to the mouth-wide mouth bottle to keep the volume constant. Before and after the experiment, the carbon source samples were analyzed by Scanning electron microscopy (SEM) and Fourier transform infrared (FT-IR) spectroscopy. The carbon sources were crushed and randomly sampled to observed using SEM (ZEISS Gemini SEM 300, Germany), and the carbon distribution on the surface of the solid carbon source was analyzed using SEM-energy dispersive X-ray spectroscopy (EDS) (ZEISS Gemini SEM 300, Germany). The functional group information of carbon sources was identified by FTIR using a FTIR spectrometer (Thermo Scientific Nicolet iS20, USA). The physical properties of the three repeated tests are shown in Table 1.

#### Nitrate removal experiment

**Figure 1:**
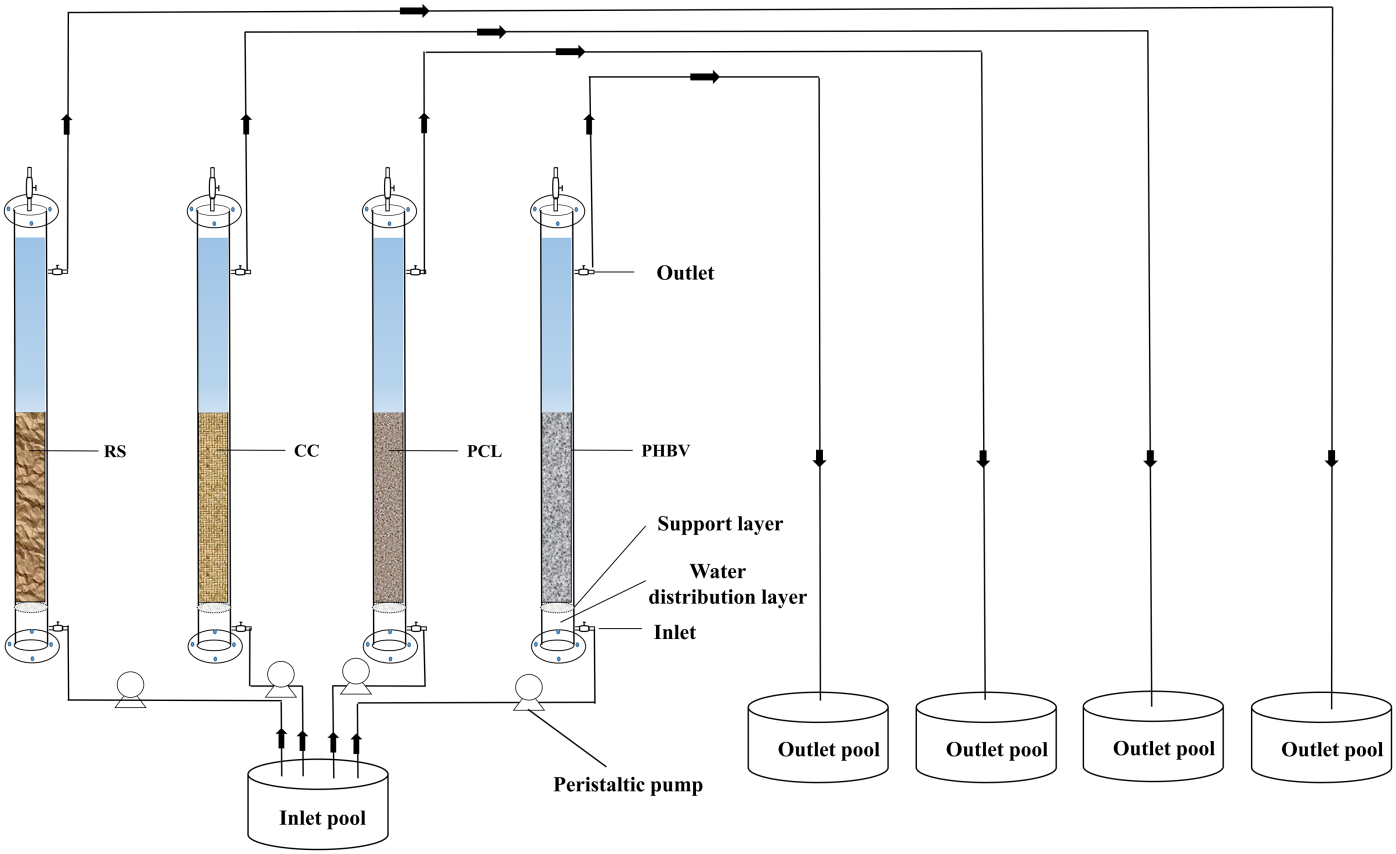
Schematic of the denitrification biofilm reactors.

The apparatus used for nitrate removal experiment is shown in Figure 1. Each experimental group included a column reactor, peristaltic pump, inlet pool (180 L) and outlet pool (10 L). The column reactor was made of completely transparent acrylic sheet, with an internal diameter of 50 mm and a height of 900 mm, and the reactor was filled with 1.2 L wastewater without adding carbon source material. The reactor was divided into a water distribution layer, a support layer (pore diameter of 3 mm, to prevent carbon sources from falling into the water distribution layer), and filling layer from bottom to top, with four outlets on the column. The experimental inlet water was artificially simulated by recirculating mariculture wastewater with a salinity of 31 ± 1 ‰, NO_3_^−^-N concentration of 30 mg/L (adjusted by KNO_3_), phosphate (PO_4_^3−^-P) concentration of 1 mg/L (adjusted by KH_2_PO_4_), pH of 7.0–7.5 (adjusted by HCl), and the temperature was controlled at 25 ± 1°C during the experiment.

In order to improve the membrane hanging start-up efficiency of the reactor, the denitrifiers adapted to high salinity were acclimated and enriched before the experiment, and detailed steps are as follows: solid wastes discharged from marine recirculating aquaculture system were used as seeding sludge and inoculated into nutrient solution (30 mg/L NO_3_^−^-N, 1 mg/L PO_4_^3−^-P, salinity 32‰), and reached mixed liquor suspended solids (MLSS) for 3 g/L. MLSS concentrations were measured according to the Standard Methods (APHA et al., 2012). Before the experiment, four types of carbon source materials were added into the reactor, and then seeding sludge was evenly inoculated into the column reactor. The carbon source fill rate was 50% and the hydraulic retention time (HRT) was set to 5 h. The NO_3_^−^-N and NO_2_^−^-N content in the outlet were sampled and measured, and the start-up operation finished as the biofilm reactor reached a steady-state with the effluent NO_3_^−^-N less than 1.5 mg/L, and this process lasts about 2 months.

The experimental phase was 180 h. The wastewater in the inlet pool was pumped into the inlet through a peristaltic pump and discharged from the outlet to the outlet pool through the reactor. The NO_3_^−^-N, NO_2_^−^-N, NH_4_^+^-N, total nitrogen (TN), and COD concentrations at the outlet were measured every 8 h. Water samples were filtered first using a 0.45 μm membrane, and then NO_3_^−^-N, NO_2_^−^-N, TN (no membrane filtration), and NH_4_^+^-N were determined using an automatic nutrient analyzer (QuAAtro, SEAL, Germany). The nitrate removal efficiency (NRE) was calculated as follows:


(1) $$NRE=\frac{{\left(NO_{3}^{-}-N\right)}_{\inf}-{\left(NO_{3}^{-}-N\right)}_{eff}}{{\left(NO_{3}^{-}-N\right)}_{\inf}}\times 100\%$$

### Bacterial community

Four carbon sources were taken for 16S rRNA high-throughput sequencing after the nitrate removal experiment. Microbial community genomic DNA was extracted from the carbon sources using the FastDNA® Spin Kit for Soil (MP Biomedicals, Norcross, GA, U.S.) according to the manufacturer’s instructions. The hypervariable region V5-V7 of the bacterial 16S rRNA gene was amplified with primer pairs 799F (5’-AACMGGATTAGATACCCKG-3′) and 1193R (5’-ACGTCATCCCCACCTTCC-3′) using an ABI GeneAmp® 9700 PCR thermocycler (ABI, CA, USA) (Bulgarelli et al., 2012). In order to analyze the structure of microbial community, the Illumina MiSeq sequencing was conducted at the Shanghai Majorbio Bio-pharm Biotechnology Co., Ltd. (Shanghai, China). The raw reads were deposited in the NCBI Sequence Read Archive (SRA) database (Accession Number: PRJNA864076).

Operational taxonomic units (OTUs) were clustered with a 97% similarity cutoff (Stackebrandt and Goebel, 1994; Edgar, 2013) using UPARSE version 7.1(Edgar, 2013), and chimeric sequences were identified and removed. The taxonomy of each OTU representative sequence was analyzed using the RDP Classifier version 2.2 (Wang et al., 2007) against the Silva v138 16S rRNA database using a confidence threshold of 70%. Detailed method has been in Supplementary material Data Sheet 1.

### Abundance of functional genes

The remaining DNA samples used for sequencing in Section 2.3 were used for quantitative real-time PCR (q-PCR) analysis. The key genes, including *narG* (encoding the membrane-bound nitrate reductase), *napA* (encoding the periplasmic nitrate reductase), *nirK* (encoding the copper-containing nitrite reductase), *nirS* (encoding the haem-containing nitrite reductases), *norB* (encoding nitric oxide reductase), and *nosZ* (encoding nitrous oxide reductase), were further quantified by q-PCR using a ChamQ SYBR Color qPCR Master Mix (2X) with an ABI PRISM 7300 Sequence Detection System (Applied Biosystems, USA), and were conducted in triplicate in different experimental groups. Each PCR tube (20 μl) contained 10 μl 2X ChamQ SYBR Color qPCR Master Mix (Nanjing Novizan Biotechnology Co., LTD, China), 2 μl DNA, 0.8 μl each of forward and reverse primer, 0.4 μl ROX Reference Dye II (50×), and sterile ddH_2_O to a total volume of 20 μl. The primers used for the PCR amplification of each gene are listed in Table 2.

**Table 2:** Primers for quantitative real-time PCR assays.

Functional gene	Primer	Primer sequence (5′-3′)	Amplification size (bp)	Source
*narG*	LIY-narGf	TCGCCSATYCCGGCSATGTC	173 bp	Chen et al. (2012)
	LIY-narGr	GAGTTGTACCAGTCRGCSGAYTCSG		
*napA*	napAF	AAYATGGCVGARATGCACCC	518 bp	Li et al. (2022)
	napAR	GRTTRAARCCCATSGTCCA		
*nirK*	nirKR3CuR	GCCTCGATCAGRTTGTGGTT	473 bp	Palmer et al. (2011)
	nirK1aCuF	ATCATGGTSCTGCCGCG		
*nirS*	cd3aF	GTSAACGTSAAGGARACSGG	400 bp	Palmer et al. (2011)
	R3cdR	GASTTCGGRTGSGTCTTGA		
*norB*	LIY-norBf	AAATGGCTTTACGTCATCGTCG	313 bp	Jiang et al. (2019)
	LIY-norBr	TCTGCGTGCCGTGGGTCT		
*nosZ*	nosZ2F	CGCRACGGCAASAAGGTSMSSGT	267 bp	Henry et al. (2006)
	nosZ2R	CAKRTGCAKSGCRTGGCAGAA		

### Statistical analysis

Statistical analysis and plotting were performed using Origin 2018 software (Origin Lab Corporation, USA). For SCFAs and gene abundance, one-way ANOVA combined with a t-test (*p* < 0.05) was conducted using SPSS Statistics 26 (SPSS, Chicago, IL, USA) to detect significant differences between the systems.

## Results and discussion

### Carbon release performance

#### Organic carbon release capacity

**Figure 2:**
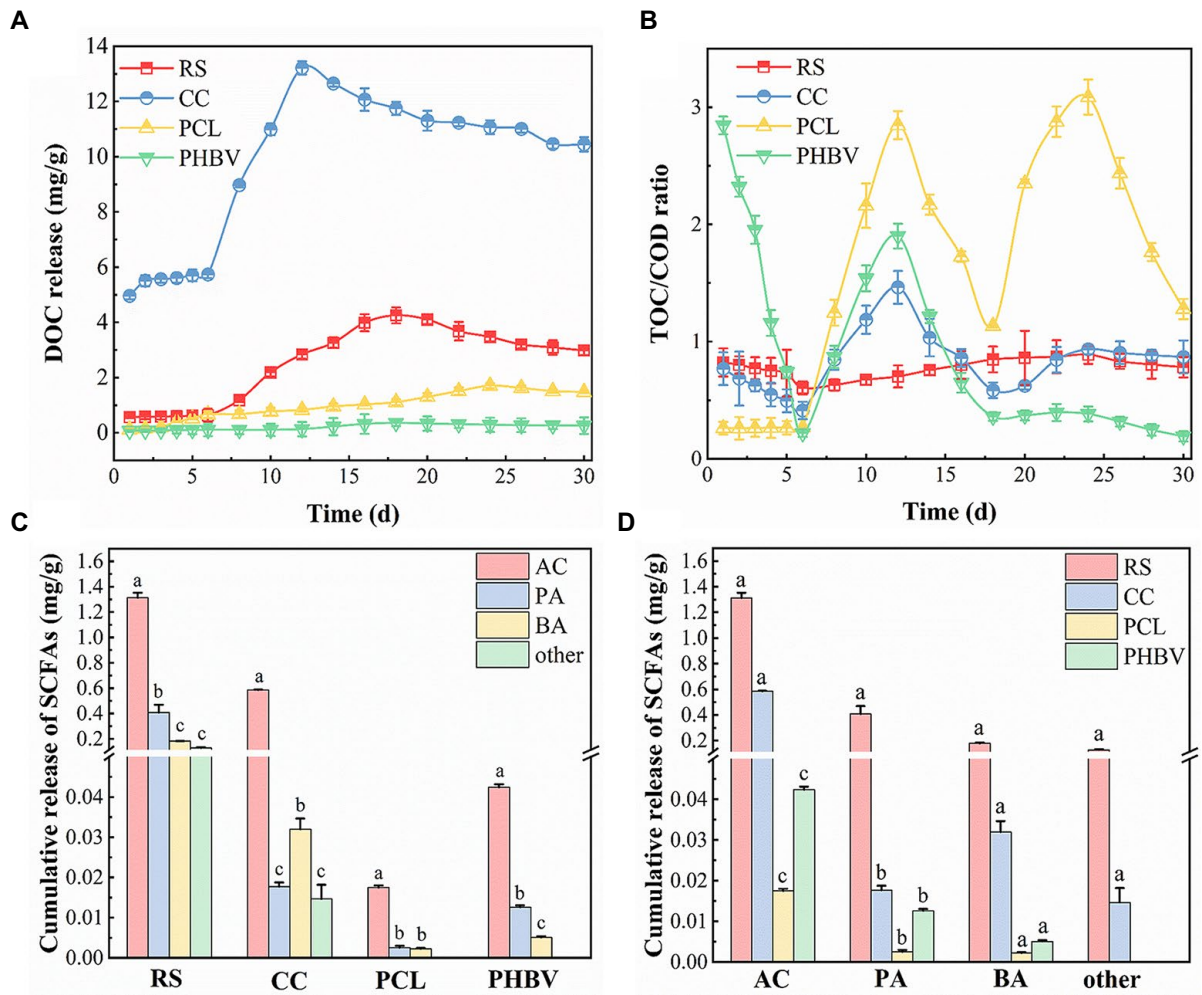
Release of different carbon sources. **(A)** Dissolved organic carbon (DOC); **(B)** Total organic carbon/Chemical oxygen demand (TOC/COD); **(C)** Differences in Short-chain fatty acids (SCFA) of the same carbon source; **(D)** Cumulative release of same SCFA with different carbon sources.

As can be seen from Figure 2A, the carbon sources of the four groups all showed an increasing trend in the early stage of the experiment, while the DOC content of the four experimental groups all decreased and tended to be stable in the later stage. The DOC contents of agricultural waste CC and RS were much higher than those of PCL and PHBV, and the average contents were CC (9.34 mg/g), RS (2.32 mg/g), PCL (0.92 mg/g), and PHBV (0.19 mg/g). Xiong et al. (2020) also observed the phenomenon of low DOC content in PCL. This is because agricultural wastes are natural cellulosic materials that contain a large number of water-soluble small molecules on the surface and inside of the materials (Zhao et al., 2020). Therefore, carbon from RS and CC was released rapidly in the early stage of the experiment under the action of the concentration difference, and the increase in the release rate was more obvious than that of PCL and PHBV. Wu et al. (2012) suggested that the growth of microorganisms affects the biodegradation of polymers, thus affecting the release of DOC. The rapid increase in DOC content due to the release of soluble products during microbial metabolism (Chu and Wang, 2013). As the carbon release experiment proceeded, the biofilm attached to the carbon source stabilized and matured gradually, showing that the content of DOC was gradually stable. In addition, higher content of DOC is beneficial to microbial growth (Chu and Wang, 2016). In the later stage, the internal concentration of the immediately releasing small molecules was low, and the remaining cellulose and hemicellulose were difficult to dissolve in water, decomposing slowly under the action of microorganisms, resulting in a decreased carbon release rate gradually. Compared to agricultural wastes, BDPs have a higher degree of polymerization and a smaller surface area, which are not conducive to microbial growth; therefore, the DOC content is low.

Agricultural waste rapidly released carbon sources in the early stage, showing that their COD contents were much higher than those of BDPs. TOC refers to the total organic carbon content in water and can be used to express the organic carbon release rate from materials into the water. The TOC/COD ratio in the leaching solution could more directly indicate the release of organic carbon from agricultural waste (Xiong et al., 2020). Carbon source release results are mainly measured by DOC index, and TOC/COD can evaluate carbon source release performance from different angles. The TOC/COD levels of the leached solutions of the four carbon sources within 30 days are shown in Figure 2B. The average ratios decreased in the order of PCL, PHBV, CC, and RS, which were 1.48, 0.98, 0.81, and 0.77, respectively. Contrary to the DOC contents, TOC/COD in the leaching solution of BDPs was higher, indicating that PCL and PHBV had higher carbon release utilization than agricultural waste. This is because agricultural waste is more likely to expand after soaking, and the small molecules inside are more likely to be released into water, resulting in a higher COD content and lower TOC/COD ratio. However, PCL and PHBV have higher degrees of polymerization, thus slower release of internal substances, lower COD concentrations, and higher TOC/COD. The use of agricultural waste-based carbon sources is relatively low, and there is a risk of exceeding COD standards and wasting carbon sources. This phenomenon was also reported by Ling et al. (2021).

#### SCFAs components of released carbon

SCFAs are commonly used as nitrogen and carbon sources for denitrification (Atasoy et al., 2018; Xiong et al., 2019; Wang et al., 2021). Different SCFA contents and combinations had different effects on denitrification performance. In this experiment, SCFAs from four carbon sources were measured (Figures 2C,D). The results showed that the contents of AC, PA, BA, and other SCFA in agricultural waste were higher than those of BDPs (Figure 2D). The cumulative released amounts of SCFAs in descending order is as follows: RS (2.08 mg/g) > CC (0.65 mg/g) > PHBV (0.06 mg/g) > PCL (0.02 mg/g). In agricultural waste, the AC content of RS (1.330 mg/g) was significantly higher than that of CC (0.588 mg/g), whereas, in BDPs, the AC content of PHBV (0.043 mg/g) was higher than that of PCL (0.018 mg/g). Reports have pointed out that when using acetate as a carbon source and electron donor, nitrate and nitrite reduction rates are faster, and AC has a simple biodegradation pathway and can be directly used in denitrifying bacterial systems (Constantin and Fick, 1997). The carbon source with a higher AC content is more conducive to denitrifying bacteria transformation and decomposition (Elefsiniotis et al., 2004), and nitrite is reduced simultaneously when acetate remains sufficient. When the AC content is insufficient, nitrate can be converted into nitrite due to the influence of substrate competition.

However, the BA content is also a factor affecting denitrification. In descending order, the BA contents in RS, CC, PHBV, and PCL were 0.18, 0.03, 0.005, and 0.002 mg/g, respectively. In the agricultural waste, RS contained much more BA than CC, which was six times that of CC. PHBV contained 2.5 times more BA than PCL in the BDPs. These results suggest that RS and PHBV may limit denitrification efficiency because of their high BA content. The PA content was similar to that of the BA. Studies have found that the removal efficiency of nitrate is very low when only butyrate is used as a carbon source (Li et al., 2015). Chen et al. (2016) found that when butyrate content increased from 5% to 30%, the NRE decreased from the highest value of 95.75% to the lowest of 42.5%. This indicates that a high concentration of BA inhibits nitrate removal, resulting in a reduced denitrification efficiency.

Chen et al. (2016) also found that a higher concentration of propionate leads to an excessive accumulation of NO_2_^−^-N during denitrification. When the acetate/propionate ratio changed from >1 to <1, the accumulation of nitrite increased by 5 mg/L. The acetate/propionate ratios of the four carbon sources were > 1, among which that of CC was the first (33.57), followed by PCL (7.00), PHBV (3.40), and RS (2.99). Therefore, the use of CC and PCL may produce less NO_2_^−^-N.

#### Surface characteristics of carbon source

**Figure 3:**
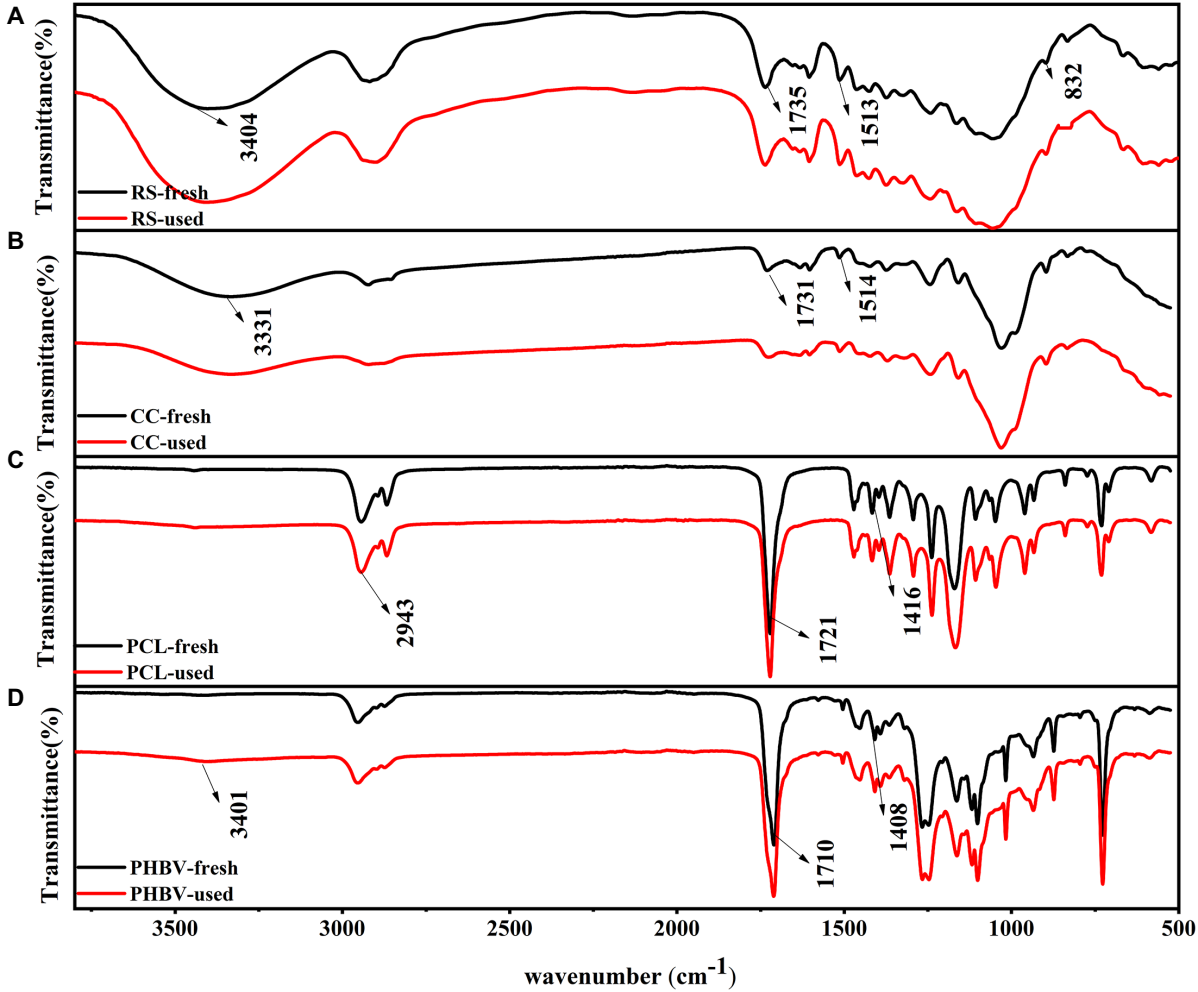
Comparison of FT-IR spectra of fresh and used carbon sources. **(A)** RS; **(B)** CC; **(C)** PCL; **(D)** PHBV.

**Figure 4:**
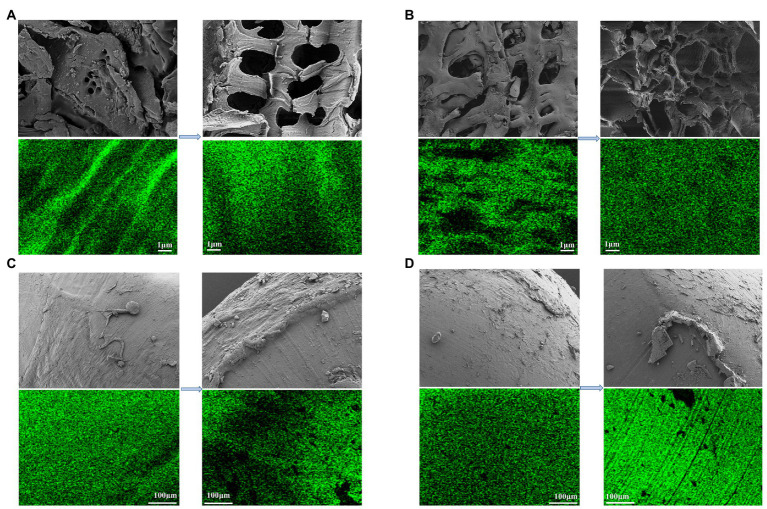
Distribution of SEM images and C of each carbon source before and after carbon released. **(A)** RS; **(B)** CC; **(C)** PCL; **(D)** PHBV.

Changes in the molecular bonds and functional groups were detected by FT-IR spectroscopy before and after the carbon source release experiment (Figure 3). FT-IR showed the organic components in different carbon sources at the functional group level. The comparison of the functional groups between agricultural waste and BDPs was quite distinct. BDPs contained more C single bonds and more complex spectra than agricultural waste. However, the FT-IR spectra of the carbon sources of the same type were similar, this suggests the presence of similar organic components in the same type of carbon source, which is similar to the results of Liang et al. (2009). The strong absorption peaks of fresh RS and CC at 3404 and 3,331 cm^−1^ were assigned to the N-H stretching vibration, and the peak shape pair was wider (Figures 4A,B), indicating that RS and CC contained many free hydroxyl groups. The absorption peaks of RS, CC, PCL, and PHBV at 1,735, 1,731, 1,721, and 1,710 cm^−1^ represent C=O stretching (Wei et al., 2012), among which PCL and PHBV had the strongest absorption peaks. The absorption peaks of RS and CC at 1,513 and 1,514 cm^−1^, respectively, belong to amide bands I and II of cellular proteins (Munir and Jamil, 2018). The absorption peaks of fresh RS and CC at 1,374 cm^−1^ were attributed to the bending vibration of-CH3. Comparing the fresh and used carbon sources, the spectral morphology of FT-IR between RS and CC is more similar than that between PCL and PHBV, RS and CC with little change in peak position. However, the weak peak at 832 cm^−1^ disappeared in RS-used, indicating that many ring structures of fresh RS were destroyed during the carbon release experiment. The spectral shapes of fresh PCL and PCL-used samples were similar (Figures 3C,D), and the shapes and positions of the characteristic peaks did not change remarkably. PCL-used showed a decline in peak at 2,943 cm^−1^, and a peak of PHBV-used at 3,401 cm^−1^ belonged to the weak peak O-H, showing that metabolites containing hydroxyl groups were generated as a result of hydrolytic degradation (Chu and Wang, 2011). The significant peaks of PCL and PHBV located at 1,416 and 1,408 cm^−1^, respectively, indicate the presence of proteins, and Guo et al. (2022) also observed the same phenomenon. When using PCL as carbon source, Chu and Wang (2011) found that the effluent contained proteins and soluble microbial by-products, namely organic compounds derived from microbial metabolites.

Figure 4 shows the SEM images of the four carbon sources and the distribution of C before and after the carbon source release experiment (Since BDPs cannot be observed clearly at 5, 100 μm was chosen). The surfaces of agricultural waste were rougher than those of BDPs, with more convex and porous structures, both before and after the experiment. This is consistent with the results of the specific surface area and pore volume of the fresh carbon sources, which were higher for agricultural waste than for BDPs (Table 1). Carbon sources with a larger specific surface area may accelerate the denitrification process and allow more bacteria to attach to it, as reported in the study by Ovez (2006). The surfaces of the PCL and PHBV were relatively smooth (Figures 4C,D). The pore structure and pore size of RS and CC increased significantly after carbon source release (Figures 4A,B). Gao et al. (2022) also observed the same phenomenon. This change is caused by the fact that agricultural waste contains crude fiber or lignin, which makes it difficult to degrade, and provides more pore structure with soluble substances, which may also be the reason for the large amount of carbon released from agricultural waste. The structure of RS-used is slightly loose, but the surface structure of CC-used is uniform and relatively compact, and still maintains a stable physical structure, which indicates that the RS structure is not strong enough. The PCL and PHBV showed significant traces of corrosion shedding and partial protrusions; similar to the result observed by Chu and Wang (2013). The green dots represent the elemental C on the surface of the carbon source. It can be argued that, compared to the fresh carbon sources, the distribution intensity of the C element in the carbon source post release increased to varying degrees. Fresh RS and CC accounted for 45.71% and 65.72%, respectively of the total atomic distribution, which significantly increased to 77.62% and 72.90%, respectively, post release. The atomic ratios of PCL-fresh and PHBV-fresh C were 76.04% and 73.44%, respectively, which increased to 79.13% and 76.51%, respectively, post release. RS and CC were more densely distributed C elements than PCL and PHBV, possibly because RS and CC were partially decomposed during carbon release, resulting in an increased surface area (Zhang et al., 2007).

### Operation performance of denitrification process based on carbon sources

#### Nitrogen removal performance

**Figure 5:**
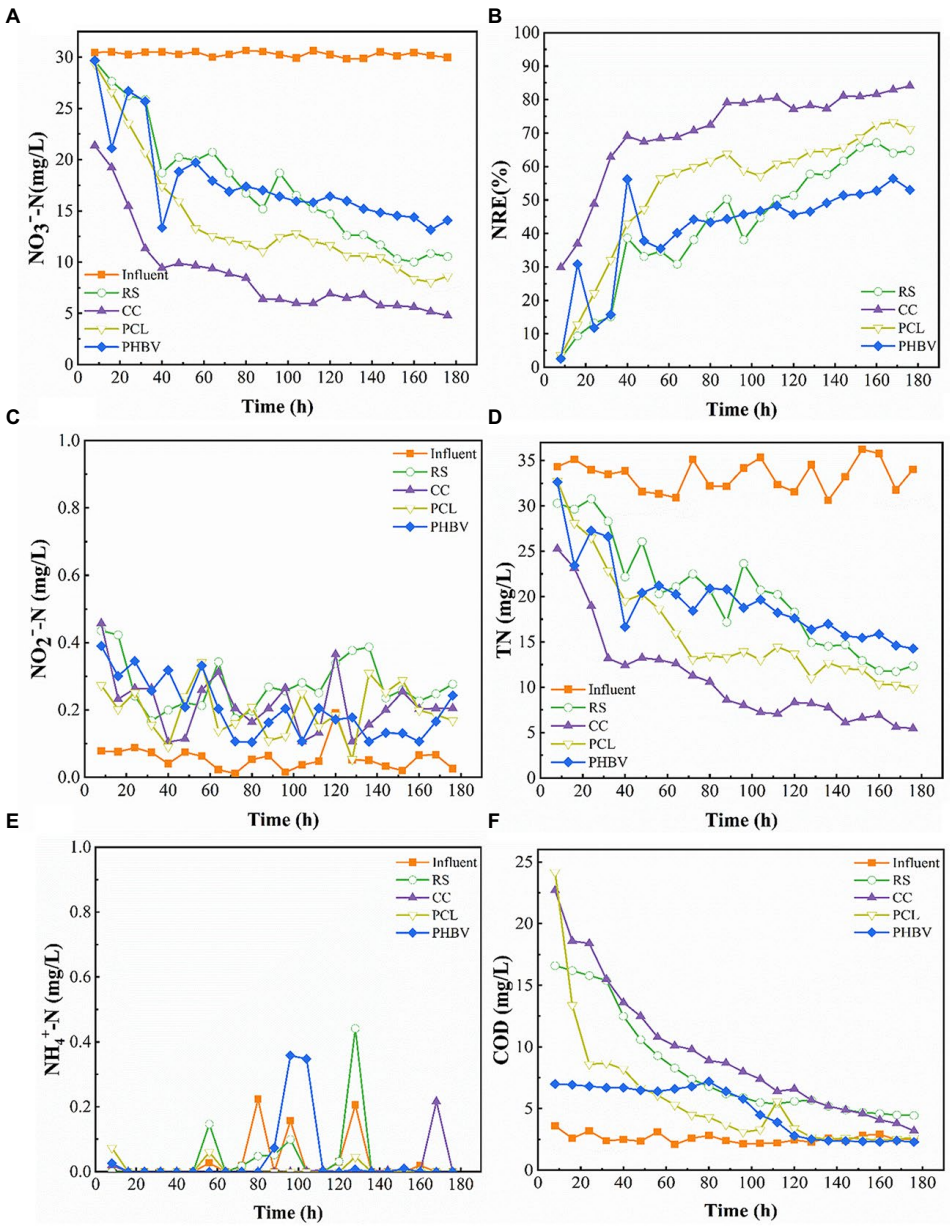
Nitrate removal performance of different carbon sources. **(A)** NO_3_^−^-N concentrations; **(B)** Nitrate removal efficiency (NRE); **(C)** NO_2_^−^-N accumulation; **(D)** Total nitrogen (TN) concentrations; **(E)** NH_4_^+^-N accumulation; **(F)** Chemical oxygen demand (COD) accumulation.

The nitrate removal performance of the continuous flow systems filled with different carbon sources within 180 h is shown in Figure 5. During the entire experiment, influent NO_3_^−^-N remained at approximately 30 mg/L, while the effluent NO_3_^−^-N concentration exhibited a downward trend, indicating that all four carbon sources could provide electron donors for the denitrification process (Figure 5A). The average NREs of CC, PCL, RS, and PHBV was 70.80%, 53.64%, 42.51%, and 41.35%, respectively (Figure 5B), CC had the highest denitrification efficiency. The lower NRE of RS and PHBV may be related to their high butyrate levels, which limit the denitrification rate (Figure 2C). The FT-IR (Figure 3A) and SEM (Figure 4A) results also indicated that the structure of RS was damaged after the carbon source release experiment, which could lead to its lower NRE. The high NRE results for CC and PCL corresponded to their higher DOC release in the immersion experiment (Figure 2A). In all experimental groups, the NRE showed a trend of a rapid increase in the first 40 h, followed by a decreasing trend during 40–160 h, and high NRE was maintained from 160 h to 180 h. The maximum NRE of the four reactors were RS 67.13% (160 h), CC 84.12% (176 h), PCL 73.26% (168 h) and PHBV 56.39% (168 h), respectively. CC always had the highest NRE within 180 h, this may be because the CC structure was more compact and rich in pore structures as indicated by SEM (Figure 4C). This indicated that CC was more conducive to the adaptation and sustainable action of denitrifiers in the environment. In addition, the NRE of CC reached more than 60% faster than that of several other systems (CC 24 h; PCL 80 h; RS 144 h; and PHBV never reached). This may be because the higher AC content and lower butyrate content after CC immersion were more conducive to improving denitrification efficiency. In the RS and PHBV systems, the residual amount of NO_3_^−^-N increased, and the NRE removal trend fluctuated during the experiment, which may be partly due to the partial disintegration of RS and PHBV during use. The SEM results (Figures 4A,D) also confirmed this result. Corrosion of RS and PHBV also affects microbial attachment and biofilm integrity, which may further inhibit denitrification rates. The disintegration of RS and PHBV will contribute to the discharge of denitrifying bacteria out of the system, affecting the continuous and efficient removal of nitrate nitrogen, which may indicate that CC and PCL have more advantages when the system is used for a long time.

The generation of NO_2_^−^-N in all the systems during the entire experimental period is shown in Figure 5C. The production of NO_2_^−^-N was lower than 0.5 mg/L, and no accumulation occurred. Among the four experimental groups, the average NO_2_^−^-N production in RS was the highest (0.274 mg/L), which may be related to the high PA content in RS (Figure 2C). NO_2_^−^-N concentration in CC was 0.217 mg/L lower than that in RS. Compared to PCL, PHBV had a slightly higher average NO_2_^−^-N residual concentration (PHBV 0.204 mg/L; PCL 0.197 mg/L), which is consistent with the results of PA in SCFAs. This corresponds to the results of the AC/BA ratio. Agricultural waste CC with a higher AC/PA ratio produced less NO_2_^−^-N than RS. This was also confirmed by the BDPs. PHBV with a lower AC/PA ratio produced more NO_2_^−^-N than PCL. The production of NO_2_^−^-N has also been reported in previous studies (Xia et al., 2022). This might be because, in the process of denitrification, nitrite reductase is inhibited due to the electron competition with NO_3_^−^-N reductase, resulting in lower respiration of nitrite and thus a lower reduction rate of nitrite (Ge et al., 2012).

A decrease in TN was observed in all four experimental groups (Figure 5D). The effluent TN content slightly differed among all four groups. NO_3_^−^-N occupies a large proportion in TN (78.86%–97.55%). The TN removal effect in the CC group was more significant (67.33%), followed by PCL, PHBV, and RS (51.23%, 41.1%, and 39.34%, respectively), which was consistent with the NRE results. As shown in Figure 5E, all four systems achieved good NH_4_^+^-N removal, and the average concentrations were < 0.01 mg/L with a descending order of PCL (73.12%) > CC (61.98%) > PHBV (34.20%) > RS (23.45%). This indicated that almost no dissimilatory nitrate reduction to ammonia occurred in any of the denitrification processes.

#### Utilization of organic matter

The COD content was high in the early stage of the experiment, and the decline rate was rapid in the first 80 h and tended to be low in the later stage (Figure 5F). Yang et al. (2015) also reported similar results. The denitrification efficiency was low at the beginning of the experiment, and the complete denitrification process was not carried out, and consequently, non-utilization of the released carbon sources led to the high accumulation of COD in the effluent water. With the growth of the denitrifying bacteria, the denitrification rate increased and the COD removal efficiency also increased rapidly. Subsequently, the systems maintained a high NRE, the release of the solid carbon source and consumption by denitrifying bacteria reached a balance, and the COD content of the effluent reached a stable state. The experimental effluent COD of the system with agricultural waste as the carbon source was higher than that of the system with BDPs. The average effluent COD content of each system was the highest with CC (9.52 mg/L), followed by RS (8.25 mg/L), PCL (5.78 mg/L), and PHBV (4.90 mg/L). The effluent COD of the four systems in the experiment was stable within the range 2.30–4.45 mg/L at the later stage of the experiment, which met the primary discharge standard of mariculture water (<10 mg/; SC/T 9103-2007, 2007).

### Microbial community analysis

#### Analysis of bacterial community diversity and structure

**Figure 6:**
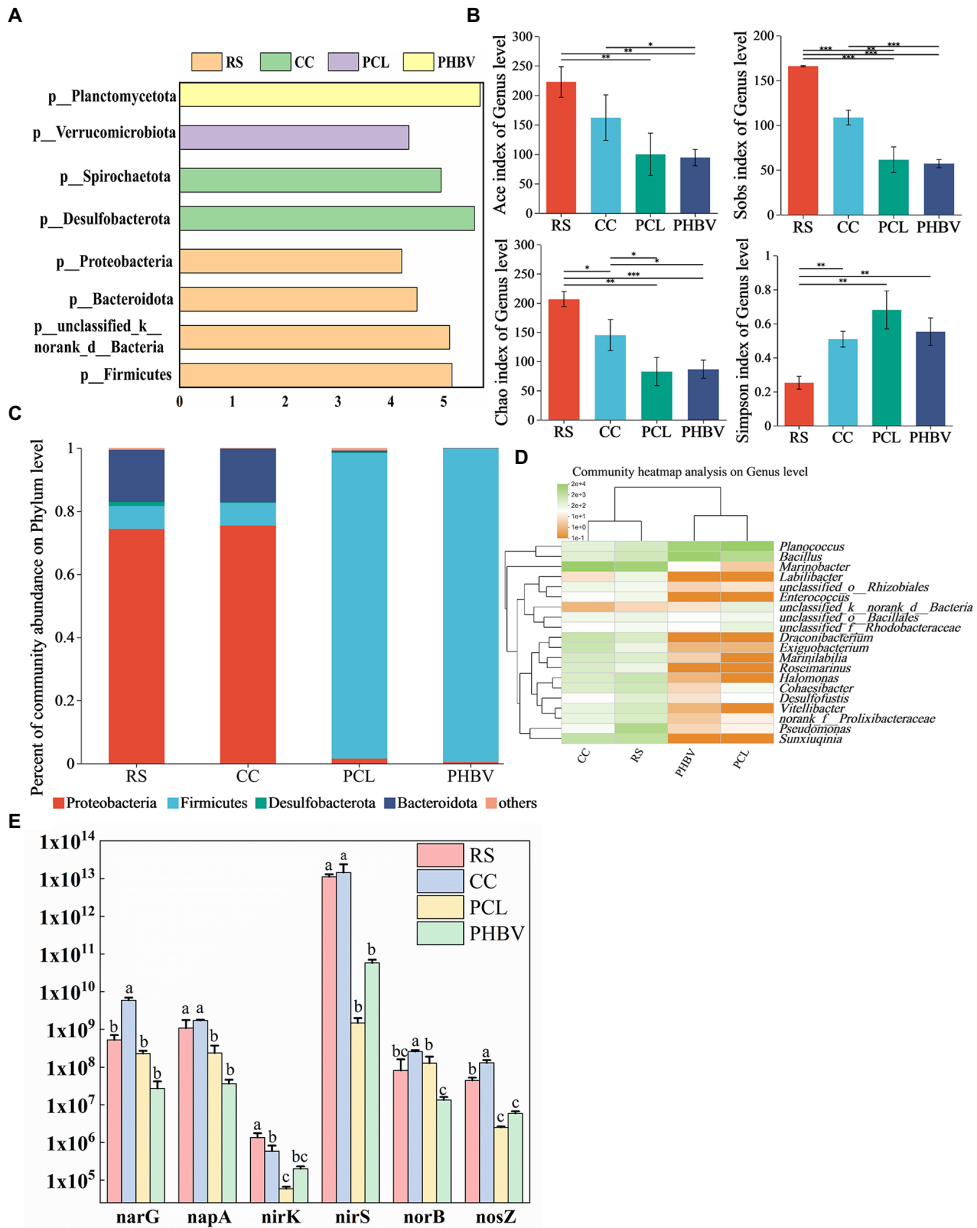
Composition and structure of microbial and abundance of nitrogen-functional genes. **(A)** The linear discriminant analysis (LDA) analysis at phylum level; **(B)** Relative abundance at phylum level; **(C)** Alpha diversity index of microbial community at genus level; **(D)** Clustering heat maps at the level of the first 20 genera; **(E)** Differences in the abundance of the same gene with different carbon sources.

The alpha diversity index results are shown in Figure 6B. A total of 58,054–60,965 effective sequences were obtained after the quality control process of raw sequence data (RS 60635, CC 58054, PCL 59365, and PHBV 60965), and the coverage rates of the four samples were greater than 0.99, indicating the high reliability and representativeness of the determination results. The Sobs index showed that the number of microorganisms observed in RS and CC was higher than that in PCL and PHBV. Chao and Ace indices in agricultural waste were higher than those in BDPs, indicating that higher cell flora proliferated by the use of RS and CC, and agricultural waste had higher species richness. The Simpson index showed that the species uniformity of BDPs was higher than that of agricultural waste.

According to linear discriminant analysis (LDA), Figure 6A shows that Firmicutes, Bacteroidetes, and Proteobacteria were the dominant phyla in RS, while Desulfobacterota and Spirochaeota were the dominant phyla in CC. The dominant phyla in PCL and PHBV were the Verrucomicrobiota and Planctomycetota, respectively. The relative abundances of Proteobacteria in RS (74.39%) and CC (75.44%) were higher than those in PCL (1.59%) and PHBV (0.44%) (Figure 6B). Proteobacteria that include many denitrifying bacteria is the most important phylum in the biological denitrification system (Si et al., 2018), which might be the reason for the high NRE of agricultural wastes. Firmicutes were more abundant in PCL (96.84%) and PHBV (99.36%) than in RS (7.20%) and CC (7.11%). Some strains of Firmicutes produce extracellular enzymes that decompose biodegradable polymers (Chu and Wang, 2016), contributing significantly to hydrolysis and acidification (He et al., 2018), which might indicate that BDPs are beneficial for carbon source supplementation and nitrogen removal in denitrification. The primary difference in Proteobacteria between agricultural waste and BDPs was seen in the population of *Pseudomonas* (Figure 6D). Its contents in PCL and PHBV were 0.08% and 0.02%, respectively, which increased to 74.39% and 75.44% in RS and CC, respectively. The high *Pseudomonas* content in CC was consistent with the high NRE results of CC. Studies have shown that *Pseudomonas* can use soluble organic matter for denitrification (Mergaert et al., 2001). The difference in Firmicutes between agricultural waste and BDPs was in *Bacillus* population. Its concentrations in system with PCL and PHBV were 15.62% and 60.90%, respectively, but reduced to only 2.89% and 1.14% with RS and CC, respectively (Figure 6D). The relative abundances of Bacteroidetes in systems with RS, CC, PCL, and PHBV were 6.60, 17.07%, 0.53%, and 0.08%, respectively (Figure 6C). Studies have shown that Bacteroidetes are related to the degradation of macromolecular organic matter, such as starch, fiber, and protein (Fang et al., 2020). A higher Bacteroidetes content is beneficial for organic matter degradation (Jiang et al., 2021) and COD removal efficiency (Liang et al., 2020). These results indicate that compared to BDPs, the microbial community function in agricultural waste was more focused on nitrate removal and organic matter degradation.

#### Nitrogen functional genes of the denitrification bioreactor

Ji et al. (2012) reported that *narG* and *napA* were key functional genes involved in nitrate to nitrite transformation and participated in the first step of denitrification. *nirK* and *nirS* are the marker genes from nitrite to nitric oxide, which is the second step of denitrification. *NorB* is involved in the third step of denitrification, which is the functional gene of nitric oxide to nitrous oxide. *NosZ* is the signature gene for nitrous oxide to nitrogen, the final step in denitrification. Therefore, the q-PCR method was used to measure the absolute abundance of nitrogen functional genes in different experimental groups. The abundance of different genes varied greatly among the four carbon sources (Figure 6E). Six genes showed the highest expression in CC, among which *narG*, *norB* and *nosZ* were significantly higher than other groups. With the exception of *norB*, the other genes were expressed in RS second to CC, indicating the advantage of agricultural waste in reducing nitrate nitrogen concentration. The absolute abundance of *nosZ* in agricultural waste is higher than that of BDPs, which may be related to the high abundance of *Pseudomonas* sp. in CC and *Marinobacter* sp. in RS (Krishnani, 2010). *NorB* gene had the highest copy number in CC, higher than RS in PCL, while PHBV content was the lowest. *NirS* showed the highest abundance in the four systems, *nirK* was lower than other genes in all systems. The lower oxygen and higher organic matter in the reactor may be the reasons for the adverse growth of *nirK* (Chen et al., 2021). This may indicate that CC and PCL can reduce nitrite nitrogen accumulation in more pairs. In general, the abundance of nitrogen-functional genes with different carbon sources was consistent with the results of nitrogen removal performance among biological systems.

## Conclusion

In this study, carbon release process and denitrification performance of agricultural waste (reed straw (RS), corn cob (CC)) and biodegradable natural or synthetic polymers (BDPs) (polycaprolactone (PCL) and poly3-hydroxybutyrate-hydroxypropionate (PHBV)) in synthetic marine recirculating quaculture wastewater were studied comprehensively. Agricultural waste released more organic matter than BDPs, and acetic acid was the largest component of the SCFA release. The four carbon sources can significantly enhance denitrification, and CC has the strongest denitrification removal effect. The primary microbial community in agricultural waste was Proteobacteria, whereas in BDPs, it was Firmicutes. Among the six genes, *nirS* and *nirK* were the most abundant and least abundant functional genes among the four carbon sources, respectively. Therefore, CC is found to be more feasible as additional carbon source for denitrification treatment of marine recirculating aquaculture wastewater.

## Data availability statement

The datasets presented in this study can be found in online repositories. The names of the repository/repositories and accession number (s) can be found at: https://www.ncbi.nlm.nih.gov/genbank/, SRP864076.

## Author contributions

YF: investigation, writing-original draft, and data curation. LW: resources, writing-review and editing. ZY: investigation and formal analysis. ZC: conceptualization, methodology, and funding acquisition. KQ: methodology, writing-review and editing. DW: software and data curation. ZW: methodology and resources. SZ: formal analysis and data curation. HC: software, writing-original draft, writing-review and editing. All authors contributed to the article and approved the submitted version.

## Funding

This work was supported by the National Key Research and Development Program of China (2020YFD0900603 and 2020YFD0900600); Central Public-interest Scientific Institution Basal Research Fund, CAFS (No. 2020TD49 and 2021XT0604); and Marine S&T Fund of Shandong Province for Pilot National Laboratory for Marine Science and Technology (Qingdao) (No.2021QNLM050103-3).

## Conflict of interest

The authors declare that the research was conducted in the absence of any commercial or financial relationships that could be construed as a potential conflict of interest.

## Publisher’s note

All claims expressed in this article are solely those of the authors and do not necessarily represent those of their affiliated organizations, or those of the publisher, the editors and the reviewers. Any product that may be evaluated in this article, or claim that may be made by its manufacturer, is not guaranteed or endorsed by the publisher.

## Supplementary material

The Supplementary material for this article can be found online at: https://www.frontiersin.org/articles/10.3389/fmicb.2023.1141362/full#supplementary-material
